# Association of the serotonin transporter-linked polymorphic region genotype with lower bone mineral density

**DOI:** 10.1038/tp.2017.184

**Published:** 2017-08-22

**Authors:** M I Lapid, S Kung, M A Frye, J M Biernacka, J R Geske, M T Drake, M D Jankowski, B L Clarke

**Affiliations:** 1 Department of Psychiatry and Psychology, Mayo Clinic, Rochester, MN, USA; 2 Department of Health Sciences Research, Division of Biomedical Statistics and Informatics, Mayo Clinic, Rochester, MN, USA; 3 Department of Medicine, Division of Endocrinology, Diabetes, Metabolism, and Nutrition, Mayo Clinic, Rochester, MN, USA; 4 Department of Information Technology, Mayo Clinic, Rochester, MN, USA

## Abstract

The serotonin transporter-linked polymorphic region (5-HTTLPR) of the serotonin transporter gene (*SLC6A4*) S allele is linked to pathogenesis of depression and slower response to selective serotonin reuptake inhibitors (SSRIs); depression and SSRIs are independently associated with bone loss. We aimed to determine whether 5-HTTLPR was associated with bone loss. This cross-sectional study included psychiatric patients with both 5-HTTLPR analysis and bone mineral density (BMD) assessment (hip and spine *Z*-scores if age <50 years and *T*-scores if ⩾50 years). BMD association with 5-HTTLPR was evaluated under models with additive allele effects and dominant S allele effects using linear regression models. Patients were stratified by age (<50 and ⩾50 years) and sex. Of 3016 patients with 5-HTTLPR genotyping, 239 had BMD assessments. Among the younger patients, the S allele was associated with lower *Z*-scores at the hip (*P*=0.002, dominant S allele effects; *P*=0.004, additive allele effects) and spine (*P*=0.0006, dominant S allele effects; *P*=0.01, additive allele effects). In sex-stratified analyses, the association of the S allele with lower BMD in the younger patients was also significant in the subset of women (*P*⩽0.003 for both hip and spine BMD under the additive allele effect model). In the small group of men younger than 50 years, the S allele was marginally associated with higher spine BMD (*P*=0.05). BMD *T*-scores were not associated with 5-HTTLPR genotypes in patients 50 years or older. The 5-HTTLPR variants may modify serotonin effects on bone with sex-specific effects.

## Introduction

Depressive disorders were the second leading cause of years lived with disability in 2010, accounting for considerable worldwide disability.^1^ In the United States, in 2009 through 2012, antidepressants were the third most prescribed class of medications (9% of prescriptions), after antihyperlipidemia and analgesic medications.^2^ However, little is known about the long-term adverse effects of antidepressants despite their prevalent use. Osteoporosis is an important public health problem. In the United States, in 2000 through 2011, osteoporotic fractures in postmenopausal women accounted for >40% of hospitalizations for the composite of osteoporotic fracture, myocardial infarction, stroke and breast cancer.^3^ Depression by itself has been associated with low bone mass, independent of antidepressant use.^4^ Selective serotonin reuptake inhibitor (SSRI) antidepressants have been implicated in the development of bone loss, possibly by decreasing osteoblast function through reduced Wnt signaling secondary to increased serotonin levels.^5^

SSRIs inhibit the serotonin transporter (5-HTT) reuptake of serotonin from the synaptic cleft into the presynaptic neuron, allowing more serotonin availability for neurotransmission and resulting in fewer depressive symptoms.^6^ Polymorphisms in the 5-HTT-linked polymorphic region (5-HTTLPR) of the 5-HTT gene (*SLC6A4*) result in altered transcription of the transporter. The wild type of the 5-HTTLPR polymorphism is the long (L) allele, thought to result in normal transcription; the short (S) allele results in less efficient transcription. The S allele has been linked to slower response to SSRIs.^7, 8, 9^

Several studies have explored the role of 5-HTTLPR in depression, hip fractures, and bone mineral density (BMD), with conflicting results. In a prospective study of 23 elderly patients with hip fractures, presence of the S allele predicted depression after hip fracture.^10^ In 108 males (aged 7–17 years) who were treated with risperidone and with an SSRI for a median duration of 2.8 years, the S allele was associated with an increased risk for reduced BMD at the lumbar spine and radius.^11^ However, in a 12-week open-label clinical trial of venlafaxine (a serotonin and norepinephrine reuptake inhibitor (SNRI)) for major depression in 69 adults (aged ⩾60 years), reduced bone formation was not found in those with the S allele but was found in those with the L allele and low-expressing serotonin receptor 1B (*HTR1B*) genotypes.^12^ In a cross-sectional study of 186 patients with normal BMD and 89 with osteoporosis (aged 47–65 years), no association was found between 5-HTTLPR and osteoporosis, but a significant association was found between osteoporosis and another polymorphic region of 5-HTT in intron 2 that consists of variable number tandem repeats (VNTRs; 5-HTTVNTR).^13^

Previous investigations in mouse models have given insight into the mechanism(s) by which SSRI treatment might cause bone loss. Warden *et al.*^14^ showed that mice with homozygous mutations in the gene encoding the serotonin transporter had reduced bone mass, altered skeletal microarchitecture, and inferior mechanical properties and that growing homozygous wild-type mice treated with fluoxetine had impairment of bone mineral accrual. These phenotypes resulted from a reduction in bone formation without an increase in bone resorption and were not influenced by effects on skeletal mechanosensitivity or serum biochemistries.

A more recent study in mice showed that fluoxetine acts on bone remodeling through two distinct mechanisms. Ortuño *et al.*^15^ showed that fluoxetine has peripheral antiresorptive properties, directly impairing osteoclast differentiation and function through a serotonin reuptake-independent mechanism that is dependent on intracellular calcium levels and the transcription factor Nfatc1. The study showed that short-term, 3-week treatment with fluoxetine resulted in a local antiresorptive response that increased bone mass, but there was a net loss of bone with longer term, 6-week use of fluoxetine, which was mediated by a centrally triggered increase in sympathetic activity. The brain serotonin-dependent rise in sympathetic output increased bone resorption sufficiently to counteract its local antiresorptive effect, resulting in a net effect of impaired bone formation and bone loss. Further, neutralization of the central nervous system-mediated mode of action via co-treatment with the β-blocker propranolol prevented fluoxetine-induced bone loss in the treated mice while leaving the peripheral effect intact. Collectively, these findings led the authors to conclude that their result provided evidence for a dual mode of action of SSRIs on bone remodeling and suggested a therapeutic strategy to block the deleterious effect on bone homeostasis from their chronic use. It remains unclear, however, whether these findings apply to humans treated with SSRI medications.

Thus, although several studies have addressed antidepressants and bone loss, few have investigated 5-HTT genotypes. Our study explored the relationship between the 5-HTTLPR genotype and bone loss. We hypothesized that the 5-HTTLPR S allele variant is associated with bone loss in psychiatric patients.

## Materials and methods

We conducted a cross-sectional study of adult psychiatric patients seen during a 10-year period (1 January 2003, to 21 February 2013). Patients were identified through electronic search of our institutional clinical databases, and they were selected if they had completed both *5-HTTLPR* genotyping analyses and BMD assessment. Patients who gave research authorization were included in this study, which was approved by the Mayo Clinic Institutional Review Board. There were no other exclusion criteria.

Collected information included demographics, 5-HTTLPR genotype, and hip and spine BMD. Data were also included if they were available for potential confounders, such as hysterectomy, history of smoking, and selected medications. Medications of interest included estrogen replacement, bisphosphonates, raloxifene, teriparatide, SSRI antidepressants, SNRI antidepressants, tricyclic antidepressants, monoamine oxidase inhibitor antidepressants, other antidepressants and anticonvulsants. Use of the medication in the 5 years preceding the BMD assessment was recorded without the dosage or duration of use.

Pharmacogenetic testing, including 5-HTTLPR, became clinically available in our institution in 2003. Genotyping was performed by Mayo Medical Laboratories or AssureRx Health (Mason, OH, USA). The genotypes identified consisted of homozygous long/long (LL), heterozygous long/short (LS), and homozygous short/short (SS). At that time, neither laboratory genotyped for the triallelic LA/LG/S variation, in which the L allele with the G mutation at rs25531 is thought to result in gene expression similar to that produced by the short allele.^16^

The Mayo Medical Laboratories procedure extracted DNA from EDTA-preserved whole blood with an EZ1 DSP kit (Qiagen, Valencia, CA, USA). The L and S forms of 5-HTTLPR were determined with PCR amplification similar to the method described by Wendland *et al.*^17^ PCR fragment sizes were determined with an Agilent 2100 Bioanalyzer (Agilent Technologies, Santa Clara, CA, USA). A length of 530 base pairs was considered a long promoter and a length of 486 base pairs was considered a short promoter polymorphism. The rare promoter polymorphisms that were longer or shorter were resolved with this method and given a customized result. The AssureRx Health procedure was similar, except that DNA was extracted from a buccal swab.

Areal BMD measurements (in grams per square centimeter) at the lumbar spine and total hip were made by dual-energy x-ray absorptiometry (DXA) according to established clinical protocols. By using X-rays of different energy levels, DXA allows for the subtraction of soft tissue absorption from total absorption, thereby permitting determination of bone absorption specifically. DXA lumbar spine scans were evaluated according to International Society for Clinical Densitometry (ISCD) criteria. Thus, vertebrae with deformities were deleted, and the mean value for L1-L4 BMD was recalculated from the remaining vertebrae. Consistent with ISCD recommendations, DXA results for women and men 50 years or older were reported as *T*-scores (equal to the number of s.d. above or below the reference mean for a healthy 30-year-old of the same sex as the patient). For women and men younger than 50 years, DXA results were reported as *Z*-scores (equal to the number of s.d. above or below the mean for a healthy age- and sex-matched reference mean). The most recent BMD was used for patients with multiple BMD measurements during the study period.

The association of BMD with genotype was evaluated with linear regression models and assumptions of additive allele effects (coding the genotype in terms of the number of S alleles) and dominant S allele effects (LL vs LS or SS genotype) as predictors of hip and spine *T*- and *Z*-scores. Analyses were performed in strata defined by age (<50 or ⩾50 years); *T*-scores were used for patients 50 years or older, and *Z*-scores for patients younger than 50 years. Analyses were adjusted for age in models for patients 50 years or older and were repeated in sex-specific strata, with the understanding that analyses of men were underpowered because of the small sample of men with available BMD data. Potential confounders, including use of estrogen, antidepressants or anticonvulsants within 5 years before the BMD measurement; history of hysterectomy; and smoking history were evaluated using *t*-tests. *P* values were interpreted in the context of the multiple testing that was performed. While analyses of the younger and older groups are independent, analyses of hip and spine within an age group, and those under additive versus dominant models, are not independent. We therefore used an approximate 0.01 significance threshold for determining significance for the analyses of combined male/female samples. We used the same threshold for the secondary sex-stratified analyses, recognizing that these analyses are considered exploratory and should be interpreted with greater caution given the small sample sizes, particularly for males. All statistical analyses were conducted with SAS version 9.4 software (SAS Institute, Cary, NC, USA).

## Results

Of 3016 psychiatric patients with 5-HTTLPR genotyping over a 10-year period, 239 had at least 1 BMD measurement, including 217 with hip measurements and 190 with spine measurements. Of the 239 patients, 198 (82.8%) were female, 231 (97%) were white, and the mean (s.d.) age was 52.0 (14.0) years (Table 1). The overall 5-HTTLPR genotype frequencies were LL (*n*=82; 34%); LS (*n*=121; 51%); and SS (*n*=36; 15%).

The ISCD defines the normal range for BMD *Z*-scores in adults younger than 50 years to be above −2.0. Thus, the observed mean BMD *Z*-scores of −0.66 for the total hip and −0.97 for the lumbar spine are both within the normal range for adults younger than 50 years. The mean hip and lumbar spine *Z*-scores for premenopausal women were also within the normal range for women younger than 50 years.

Of the possible confounders analyzed, only SSRI use (*P*=0.01) and antipsychotic use (*P*=0.03) within 5 years before BMD measurement were associated with BMD in patients younger than 50 years, with lower hip BMD *Z*-scores in SSRI or antipsychotic users as compared to patients who did not use SSRIs or antipsychotics. Final models in patients younger than 50 years were adjusted for only SSRI use because antipsychotic use was not a significant predictor (all *P*>0.10) in the multivariable models. Estrogen use and the use of other psychotropic medications (for example, anticonvulsants and non-SSRI antidepressants) were not significant confounders.

In patients 50 years or older, the 5-HTTLPR genotype was not associated with hip or spine BMD *T*-scores in analyses assuming additive allele effects or dominant S allele effects (Table 2). However, in patients younger than 50 years, 5-HTTLPR genotype was associated with both hip and spine BMD Z-scores (hip, *P*=0.004 assuming additive S allele effects and *P*=0.002 assuming dominant S allele effects; spine *P*=0.01 assuming additive S allele effects and *P*=0.0006 assuming dominant S allele effects), with S allele carriers having lower *Z*-scores.

Sex-stratified analyses showed similar results for women as for the sample overall, with a significant association of the S allele with lower hip *Z*-scores (*P*=0.002 for additive and *P*=0.003 for dominant analyses) and spine *Z*-score (*P*=0.003 assuming additive allele effects and *P*=0.0006 for a dominant effect) in women younger than 50 years. However, in the small sample of men younger than 50 years, the S allele was marginally associated with a higher spine *Z*-score (*P*=0.05 under an additive model).

## Discussion

To our knowledge, this is the largest study to investigate the relationship between 5-HTTLPR and bone loss. Our findings suggest that the 5-HTTLPR S allele is associated with lower BMD in adults younger than 50 years. In contrast, no evidence was found for an association between the S allele and BMD in adults 50 years or older. This association between the 5-HTTLPR genotype and BMD in adults younger than 50 years was driven by the larger sample of women; in particular, sex-stratified analysis showed that premenopausal women younger than 50 years with the S allele had lower BMD compared to women younger than 50 years without the S allele. Surprisingly, an opposite association of the S allele with higher spine BMD was observed in men younger than 50 years, a finding that warrants further investigation because it was based on a small sample.

The murine study by Warden *et al.*^14^ showed that mice with homozygous mutations in the gene encoding the serotonin transporter had reduced bone mass, altered bone microarchitecture, and inferior mechanical properties, suggesting that inhibition of the serotonin transporter in adult humans may lead to bone loss by a similar mechanism. For the younger adults in this cohort treated with SSRIs, the observation that growing wild-type mice treated with fluoxetine had impairment of bone mineral accrual may also be relevant. The mouse phenotypes were attributed to a reduction in bone formation without an increase in bone resorption and were not influenced by effects on skeletal mechanosensitivity or serum biochemistries.

These observations may suggest that the 5-HTTLPR S allele is linked to lower BMD in adults or premenopausal women younger than 50 years but not in adults 50 years or older because of effects on the growing skeleton. Given that gonadal sex steroid deficiency or age-related factors are likely to be more common and perhaps correspondingly more important as causative factors for bone loss in adults 50 years or older, it is conceivable that a modest effect associated with the S allele in older adults was not detectable in this small cohort.

The biphasic mechanism causing bone loss observed in the mouse study by Ortuño *et al.*^15^ may be directly relevant to this study cohort. The antiresorptive effect seen on osteoclasts early in therapy in the mice would likely not be observable in this human study population in which participants received longer term treatment with SSRIs. The antiresorptive effect in the mice was shown to be independent of serotonin reuptake and to act instead through osteoclast intracellular calcium and the transcription factor Nfatc1. The longer term treatment effect in the mice leading to increased central nervous system sympathetic outflow and bone loss may also be operative in humans.

It is not clear whether the 5-HTTLPR S allele might also be associated with lower BMD because of decreased platelet serotonin levels. Expression of the S allele in circulating platelets would be expected to alter platelet 5-HTT function and lead to increased circulating or tissue fluid 5-HTT levels. In turn, the increased 5-HTT levels might potentially interact with the serotonin receptor on osteoblasts, resulting in inhibition of Wnt signaling, reduced osteoblast activity, decreased bone formation, and lower BMD.^18^ This effect was not seen in the mouse study by Ortuño *et al.*^15^

The medical literature on 5-HTTLPR, BMD, depression and antidepressant use is conflicting, which speaks to the complexity of these relationships. In a study of male youths treated with risperidone and an SSRI antidepressant, the S allele was associated with a higher risk for reduced BMD at the lumbar spine and radius.^11^ Those results are consistent with the results of our study, in which the S allele was associated with reduced BMD. In contrast is a study of patients 60 years or older who were treated with an SNRI antidepressant: the L allele and another polymorphism of low-expressing serotonin receptor (*HTR1B*) were associated with reduced bone formation.^12^ Notably, those results are not consistent with our findings, which identified no association of the L allele with reduced BMD in patients 50 years or older. Another study found no association of 5-HTTLPR polymorphisms with low bone density, although a polymorphism of the 5-HTTVNTR region was associated with osteoporosis.^13^ In comparison, in a population-based retrospective study of 2978 depressed and 131 912 nondepressed middle-aged patients in Taiwan, the depressed patients were 1.3-fold more likely to have osteoporosis; interestingly, depressed patients treated with antidepressants (SSRIs and non-SSRIs) were less likely to have osteoporosis.^19^ However, that study did not include any genotyping information. In contrast, our study showed that SSRI antidepressant use was associated with lower BMD hip *Z*-scores in patients 50 years or younger.

Our study does not provide conclusive evidence of the exact interrelationship between 5-HTTLPR, BMD, depression and antidepressants. While our results showed a relationship between the *5-HTTLPR* genotype and BMD, our study did not permit us to determine the mechanism by which long-term use of SSRIs affects bone health or whether β-blockers might prevent bone loss. Given the prevalence of SSRI use and the negative effect of SSRIs on bone mass, it is important to better define these associations because they may affect recommendations for long-term antidepressant use and potential treatment options to limit bone loss and fracture risk.

Limitations of this study include the relatively small sample size and the use of a cross-sectional cohort limited to patients who had both 5-HTTLPR genotyping and the BMD assessment. Genotyping was performed primarily if patients did not have a therapeutic response or if they had adverse effects from psychotropic medication; this may induce bias and limit generalizability. For the L and S alleles genotyped, only the L_A_ allele could be identified because the laboratories at that time did not test for the L_G_ allele, which has reduced activity like the S allele. We did not genotype or analyze other serotonin-related polymorphisms, such as VNTRs or serotonin receptor genes *HTR2A*/*HTR2C*. In addition, we did not ascertain whether patients underwent BMD assessment with DXA for a fracture or for screening purposes. The retrospective method did not allow us to collect information on severity of depression and antidepressant treatment (type, doses, and length of treatment) before BMD measurement. Finally, we did not have data related to other factors likely to contribute to maintenance of skeletal health, such as serum levels of calcium, phosphorus, vitamin D, and parathyroid hormone; renal function; liver function; gonadal sex steroid status; thyroid function; and urinary calcium level.

Given the inherent limitations of cross-sectional or observational studies, it remains important for future studies to be designed to more adequately assess the relationship between antidepressant use and bone density changes. A well-designed study would be prospective, with fixed inclusion and exclusion criteria, and include longitudinal follow-up for a sufficient length of time to measure changes in bone density. Until then, observational studies should not alter clinical management of depression, particularly in older adults.^20^

## Conclusions

This study suggests that 5-HTTLPR S allele is associated with lower BMD at the hip and spine, which was most evident in women younger than 50 years. Our results suggest that 5-HTTLPR variants may modify serotonin effects on bone in a sex-specific interaction. Future studies are needed to explore the utility of 5-HTT genotypes in identifying psychiatric patients who may be vulnerable to increased bone loss.

## Figures and Tables

**Table 1:** Demographic and clinical characteristics by age group

*Characteristics*	*Age <50 years (*n=*79)*^a^	*Age ⩾50 years (*n=*138)*^a^
Age, years	40 (16-49)	59 (50-90)
Hip *T*- or *Z*-score	−0.5 (−3.1 to 1.9)	−1.0 (−3.9 to 2.7)
Spine *T*- or *Z*-score	−0.6 (−3.2 to 2.9)	−0.9 (−4.4 to 1.9)
Female	66 (84)	111 (80)
White	75 (95)	135 (98)
		
*Antidepressant*^b^		
SSRI	61 (77)	98 (71)
SNRI	29 (37)	68 (49)
MAOI	0	0
TCA	19 (24)	38 (28)
Other	29 (37)	61 (44)
		
Anticonvulsant^b^	33 (42)	56 (41)
Antipsychotic^b^	30 (38)	41 (30)
Past smoker	14 (18)	34 (25)
Hysterectomy	6 (8)	7 (5)
Hormone replacement^b^	16 (20)	46 (33)

Abbreviations: MAOI, monoamine oxidase inhibitor; SNRI, serotonin-norepinephrine reuptake inhibitor; SSRI, selective serotonin reuptake inhibitor; TCA, tricyclic antidepressant.

a Continuous data are summarized as median (range); categorical data as number of patients (percentage of sample).

b Medication use refers to any prescribed treatment in the previous 5 years.

**Table 2:** Association of BMD with 5-HTTLPR genotype using additive and dominant S allele effects

	*BMD test*		*Additive S allele effects*		*Dominant S allele effects*	
	*No.*	*Sample*	*Estimate*	P*-value*	*Estimate*	P*-value*
*Age ⩾50 years*						
Hip *T-*score^a^	138	All	0.055	0.71	0.222	0.27
	111	Female	0.127	0.47	0.283	0.22
	27	Male	−0.116	0.63	0.077	0.82
Spine *T*-score^b^	117	All	0.044	0.82	0.308	0.21
	96	Female	0.032	0.89	0.291	0.30
	21	Male	0.098	0.74	0.404	0.33
						
*Age <50 years*						
Hip *Z*-score^b^	79	All	−0.378	0.004	−0.614	0.002
	66	Female	−0.484	0.002	−0.700	0.003
	13	Male	0.151	0.41	0.141	0.68
Spine *Z*-score^b^	73	All	−0.442	0.01	−0.935	0.0006
	61	Female	−0.633	0.003	−1.047	0.0006
	12	Male	0.588	0.05	0.186	0.78

Abbreviations: BMD, bone mineral density; 5-HTTLPR, serotonin transporter-linked polymorphic region.

a Adjusted for age.

b Adjusted for selective serotonin reuptake inhibitor use.
